# Franklin Festival

**Published:** 1854-01

**Authors:** 


*Franklin Festival.*—The Buffalo Printers' Union hold their second annual festival in honor of Franklin's birth-day, at the American Hotel, in this city, on Monday evening, Jan. 16, 1854.

By a liberal and charitable construction, all editors are ranked as printers—an honor to be coveted. There is so much good feeling, genuine wit, and sound sense among the disciples of Faust as a class, that we have no doubt that their reunion will be one of the pleasantest of the season. S. B. H.

